# Response: Commentary: Past, present and future of epigenetics applied to livestock breeding

**DOI:** 10.3389/fgene.2016.00101

**Published:** 2016-06-07

**Authors:** Oscar González-Recio, Miguel A. Toro, Alex Bach

**Affiliations:** ^1^Departamento de Mejora Genética Animal, Instituto Nacional de Investigación y Tecnología Agraria y AlimentariaMadrid, Spain; ^2^Departamento de Produccion Animal, Escuela Técnica Superior de Ingenieros Agrónomos, Universidad Politécnica de MadridMadrid, Spain; ^3^Department of Ruminant Production, Institució Catalana de Recerca i Estudis Avançats – Institut de Recerca i Tecnologia AgroalimentàriesCaldes de Montbui, Spain

**Keywords:** epigenetics, animal breeding, breeding programs, Lamarckian inheritance, central dogma of molecular biology

Following our recent Review article (González-Recio et al., 2015), we received correspondence by Steele (2016). We thank Dr. Steele for his comments, which provide a thorough review of his work on human immunology, which has persuaded him that “hard types of soma-to-germline transfer are ongoing at very high frequency in human immune system germlines.” His and other researchers' studies on reverse transcriptase (RT) based feedback mechanisms showed that RNA could be retrotranscripted to DNA, and it can be inserted into the mammalian germline, and therefore be transferred to the progeny.

This is an example of Weismann's barrier permeability, and relates to the modification of the Central Dogma of Molecular Biology (Crick, 1970). We believe that our article is not in disagreement with this re-formulation of the original Central Dogma, and as such we mentioned that “the Central Dogma is not a logical necessity but a fact of the inheritance system and therefore […] one might expect some exceptions,” and provide a couple of cases that have been traditionally used as didactic examples, without the intention to claim that they are the only ones. In this sense, we apologize if we have omitted Dr. Steele and other researchers' studies on these more or less rare exceptions. We tried to keep our review as short and focused as possible on the potential application of epigenetics to livestock breeding, which is explicit in the title of our article.

Furthermore, in the section of our original article entitled “The Term “Epigenetics” and Its Current Interpretation” we explicitly mention that “the field of epigenetics has grown during the last decades,” and that “there is no consensus on the current definition of the term [epigenetics].” In this sense, non-synonymous changes in the DNA sequence caused by the RT would not be considered as epigenetic modifications in the traditional definition of epigenetics (Holliday, 2006; Bird, 2007). However, it is open to debate and interpretations according to broader, but less accepted, epigenetic definitions (Deans and Maggert, 2015).

We would like to emphasize that much of the importance of epigenetic for livestock breeding resides on the transgenerational epigenetic beyond three generations, and we maintain our view on the difficulty of documenting this type of inheritance in mammals.

We encourage researchers to comment and discuss other examples of transgenerational epigenetic inheritance in mammals, specifically those beyond three generations, that we might have unintentionally omitted in this focused review.

## Author contributions

All authors listed, have made substantial, direct and intellectual contribution to the work, and approved it for publication.

### Conflict of interest statement

The authors declare that the research was conducted in the absence of any commercial or financial relationships that could be construed as a potential conflict of interest.
